# A combination of clinical balance measures and FRAX® to improve identification of high-risk fallers

**DOI:** 10.1186/s12877-016-0266-6

**Published:** 2016-05-03

**Authors:** David A. Najafi, Leif E. Dahlberg, Eva Ekvall Hansson

**Affiliations:** Department of Health Sciences, Health Sciences Centre, Lund University, Baravägen 3, 22240 Lund, Sweden; Department of Clinical Sciences, Lund University, Lund, Sweden

**Keywords:** FRAX®, Falls, Fracture, Balance

## Abstract

**Background:**

The FRAX® algorithm quantifies a patient’s 10-year probability of a hip or major osteoporotic fracture without taking an individual’s balance into account. Balance measures assess the functional ability of an individual and the FRAX® algorithm is a model that integrates the individual patients clinical risk factors [not balance] and bone mineral density. Thus, clinical balance measures capture aspects that the FRAX® algorithm does not, and vice versa. It is therefore possible that combining FRAX® and clinical balance measures can improve the identification of patients at high fall risk and thereby high fracture risk.

Our study aim was to explore whether there is an association between clinical balance measures and fracture prediction obtained from FRAX®.

**Method:**

A cross-sectional study design was used where *post hoc* was performed on a dataset of 82 participants (54 to 89 years of age, mean age 71.4, 77 female), with a fall-related wrist-fracture between 2008 and 2012. Balance was measured by tandem stance, standing one leg, walking in the figure of eight, walking heel to toe on a line, walking as fast as possible for 30 m and five times sit to stand balance measures [tandem stance and standing one leg measured first with open and then with closed eyes] and each one analyzed for bivariate relations with the 10-year probability values for hip and major osteoporotic fractures as calculated by FRAX® using Spearman’s rank correlation test.

**Results:**

Individuals with high FRAX® values had poor outcome in balance measures; however the significance level of the correlation differed between tests. Standing one leg eyes closed had strongest correlation to FRAX® (0.610 *p* = < 0.01) and Five times sit to stand was the only test that did not correlate with FRAX® (0.013).

**Conclusion:**

This study showed that there is an association between clinical balance measures and FRAX®. Hence, the use of clinical balance measures and FRAX® in combination, might improve the identification of individuals with high risk of falls and thereby following fractures. Results enable healthcare providers to optimize treatment and prevention of fall-related fractures.

**Trial registration:**

The study has been registered in Clinical Trials.gov, registration number NCT00988572.

## Background

Fall-related fractures are a problem for society, and account for significant morbidity and healthcare expenses in elderly patients [1–3]. Approximately one third of persons over 65 years of age fall each year [4, 5]. In 5–15 % of cases, a fall in an elderly person results in a fracture [6, 7]. Fractures are the most costly fall-related injuries. In the United States, fractures account for 35 % of injuries from falls but 61 % of the healthcare costs [3]. In Sweden the annual number of hip fractures is expected to double between 2002 and 2050, predicting 30,000 hip fractures in 2050 [8].

### Risk factors for falling

Several risk factors for fall-related hip fracture have been identified, such as increasing age and previous fracture [9], and older adults with multiple risk factors and low bone mineral density are at the highest risk of hip fracture [10]. Balance is also a risk factor for falls but the variety of methods of assessing balance makes it difficult to appreciate the impact of balance on fall risk [11]. However, balance deficits can predict falls [12]. Asymmetric vestibular function affects balance [13] and is overrepresented among elderly persons with hip fractures [14] and wrist fractures [15]. It is also a strong predictor of falls among the elderly [13]. The age-related changes seen in the incidence of wrist fractures differs significantly from the increase seen in hip fracture incidence [16]. A deterioration of multiple sensory receptor systems and neuro-muscular function occurs in ageing, which probably result in a decrease in ability to extend the arm to alleviate the consequences of a fall [17], a pattern that explains the age differences between wrist and hip fracture incidence.

### Fracture risk prediction tools

Since fall-related fractures are a huge problem for the society, and not seldom lethal for the affected person [18], the use of tools for the prediction of fracture seems essential. As many as 13 different tools have been identified, including between 4 and 31 clinical risk factors [19]. The most frequent used tool in research is the FRAX® algorithm which was developed by the World Health Organization, in collaboration with The University of Sheffield. FRAX® includes an online calculation tool and uses femoral neck bone mineral density, prior fractures, parental hip fracture history, age, sex, body mass index, ethnicity [not in all countries], smoking, alcohol use, glucocorticoid use, rheumatoid arthritis and secondary osteoporosis data to quantify a patient’s 10-year probability value of a hip or major osteoporotic fracture [20]. The main use of the FRAX® algorithm in clinical practice is for to identify those individuals who need a pharmacological intervention [21]. An individual’s risk of falling is not included in FRAX®, since comparable data of falls were missing when the tool was developed [22]. Quantitative adjustment of the FRAX® by including falls history does not seem to be possible currently [23]. To accomplish further fracture reduction, reduction of fall risk is required FRAX® seems to partly apprehend risk of future fall even it fall risk is not included in the tool [24]. Attempts have been made to combine fall risk instruments and FRAX® [25]. However, correlations between the instruments were weak and probably caused by the inclusion of age and sex in FRAX® and more research is therefore needed.

### Balance

Losing one’s balance, and thus falling, is often a result of incorrect weight shifting. In the prevention of falls and thereby prevention of potential fractures, a multitask approach is beneficial [26]. Such multitask approaches comprise balance measures that assess balance and functional ability. In the elderly, these performance-oriented functional tests are widely adopted [27, 28] to identify risk groups for falling.

Balance measures assess the functional ability of an individual and the FRAX® algorithm has an individual’s rate of osteopenia as the key feature. Thus, clinical balance measures capture aspects that the FRAX® algorithm does not, and vice versa. It is therefore possible that combining FRAX® and clinical balance measures can improve the identification of patients at very high fall risk. However, if there is an association between clinical balance measures and FRAX® is unclear.

Our study aim was to explore whether there is an association between clinical balance measures and fracture prediction obtained from FRAX®.

## Methods

### Participants

*Post hoc* data analysis was performed on a dataset of 82 participants aged over 50 with fall-related fractures. Data were collected in Malmö, Sweden, between Dec 2008 and Nov 2012, when 85 patients were allocated to a randomized controlled trial. Sixty eight patients completed the study and 17 interrupted [15]. In the present study, baseline measures for the total study sample is used, expect for three patients, where FRAX® data were not available. Thus, the study comprised of 82 patients (77 female, 93.9 %) 54 to 89 years of age (mean 71.4, SD = 9.1), who all had a fall-related wrist fracture. All patients lived at home, 31 with a partner and 51 by themselves. Mean value for self-rated health, measured by the EQ5D visual analogue scale [29] was 73 (standard deviation 16.6). In this study, balance measures and FRAX® data were used. The study was approved by the regional ethical review board in Lund [number 585/2008].

### Balance measures

The balance measures used have been shown in previous studies to be relevant for assessing elderly patients in risk of falling [12, 30, 31].

The balance measures were:

- Tandem stance (standing in a heel-to-toe position) with eyes open (TSEO) and Tandem stance with eyes closed (TSEC) [32–34]. The time that the participant was able to maintain this position, up to 30 s, was measured. Three attempts were allowed and the best attempt was used.
- Standing on one leg with eyes open (SOLEO) and Standing on one leg with eyes closed (SOLEC) [33]. Time up to 30 s was measured and three attempts were allowed.
- Walking in a modified figure of eight [35], where steps outside the figure were counted.
- Walking heel-to-toe in a straight line [34], where steps outside the line were counted.
- Walking as fast as possible for 30 m with one turn after 15 m [36]. Time in seconds was measured.
- Five times sit-to-stand test (FTSST) [37] where the participant sat on a stool and stood up and sat down five times while the time was measured.

### FRAX®

FRAX® algorithms calculate the 10-year probability of fracture. The output is a 10-year probability of hip fracture and the 10-year probability of a major osteoporotic fracture (value 0.00–1.00). FRAX® values were calculated using the web-based calculation tool for Sweden [20]. FRAX® value of <5 is considered as low fracture risk, value between ≥5 and <7.5 is considered as intermediate risk and value of ≥7.5 is considered as high risk [38]. The information from the questionnaire was transferred to the calculation tool.

### Statistical analysis

All data were examined visually for skewness and kurtosis and checked for normality using the Shapiro-Wilks test. For normally-distributed variables, mean and standard deviation were presented (age), and for non-normal variables, median and interquartile range (all others).

Correlation coefficients were calculated for the bivariate relationship between each of the balance tests and the FRAX score for 10-year probability for hip and major osteoporotic fractures. Spearman’s rank correlation test was used due to the non-normality of the data.

Analyses were performed using SPSS Inc., version 22.

## Results

### Participants

Characteristics of the participants and balance measures are shown in Table 1. One participant was not able to perform neither Walking heel-to-toe in a straight line nor Walking in the modified figure of eight, although could perform the remaining balance tests thus including the results in the correlation analysis.

**Table 1:** Characteristics of the participants

Variables	All (*n* = 82)
Age, mean (SD)	71 (±9)
Women, *n* (%)	77 (94)
Body Mass Index, median (IQR)	25.5 (4.75)
Currently smoking, *n* (%)	9 (11 %)
Currently using glucocorticoids, *n* (%)	5 (6 %)
Have rheumatoid arthritis, *n* (%)	0 (0 %)
Alcohol >3 standard glasses/day, *n* (%)	1 (1 %)
FRAX®_osteo_ median (IQR)	0.27 (0.19)
FRAX®_hip_, median (IQR)	0.11 (0.13)
Physically active, *n* (%)	43 (53)
Five times sit to stand, sec median (IQR)	10 (3)
Tandem stance eyes open, sec median (IQR)	30 (0)
Tandem stance eyes closed median (IQR)	10 (25)
Standing one leg eyes open, sec median (IQR)	19 (24)
Standing one leg eyes closed, sec median (IQR)	3 (3)
Walking heel to toe, steps median (IQR)	1 (3)
Walking in a figure of eight, steps median (IQR)	2 (7)
Walking as fast as possible for 30 m, sec median (IQR)	21 (7)

*SD* Standard deviation, *IQR* Interquartile range

FRAX® = 10-year probability value of a hip or major osteoporotic fracture (0.00–1.00)

Median value for TSEO was 30 s and 91.5 % of the participants could maintain the position for 30 s.

Median value for Walking heel-to-toe was 1 step and 37 % of the participants could perform the test without stepping outside the line at all.

Median value for Walking in the figure of eight was 2 steps and 38 % could perform the test without stepping outside the figure at all.

Data on “Parent fractured hip”, “Secondary osteoporosis” and “Femoral neck BMD” were missing for all cases and equated to the non-presence of each feature for study purposes [20]. Also, the “Smoking” value for one participant was missing and set to “no” and “Rheumatoid Arthritis” value for one participant was missing and set to “no”.

### Bivariate correlation analysis

**Fig. 1:**
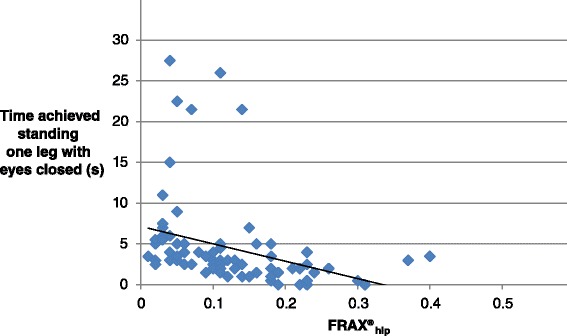
Correlation between standing one leg eyes closed and FRAX®_hip_ (Spearman’s rank correlation co-efficient = 0.61; *P* < 0.01)

**Fig. 2:**
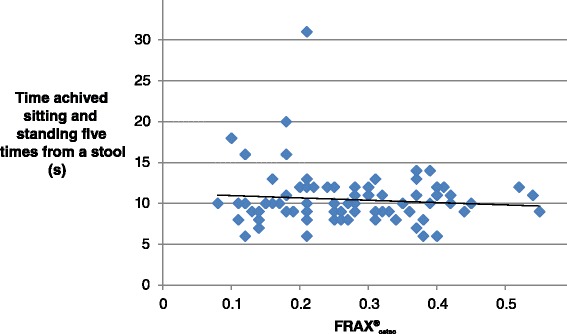
Correlation between five times sit to stand and FRAX®_osteo_ (0.01)

The bivariate correlation analysis showed that individuals with high values on FRAX® (both FRAX®_hip_ and FRAX®_osteo_) also had poor outcome on: TSEO, TSEC, SOLEO, SOLEC, Walking heel to toe, Walking in the modified figure of eight and Fast walking test (Spearmans rho −0.319–0.610). Correlations are displayed in Table 2. SOLEC had the strongest correlation with FRAX® (Fig. 1) and FTSST had no correlation to FRAX® (Fig. 2).

**Table 2:** Spearman correlation coefficients between balance measures and FRAX® osteoporotic and FRAX® hip fracture scores

Balance measures	Frax®_osteo_	Frax®_hip_
Five times sit to stand	0.01	0.04
Tandem stance eyes open	−0.32^a^	−0.34^a^
Tandem stance eyes closed	−0.43^a^	−0.44^a^
Standing one leg eyes open	−0.50^a^	−0.49^a^
Standing one leg eyes closed	−0.59^a^	−0.61^a^
Walking heel to toe	0.42^a^	0.42^a^
Walking in a figure of eight	0.51^a^	0.50^a^
Walking as fast as possible for 30 m	0.40^a^	0.40^a^

^a^Correlation is statistically significant at the 0.01 level (2-tailed)

## Discussion

### Main findings

This study indicated significant correlations between FRAX® values and seven out of eight balance measures. SOLEC had the strongest correlation to both FRAX®_osteo_ and FRAX®_hip_. These findings imply a relation between FRAX® and all clinical balance measures except FTSST in a population with high risk fallers who already had a fall-related fracture.

### Discussion of the results

Maintaining balance involves a complex system of skills in motor function, integrated with somatosensory input and processed in the brainstem. Furthermore, balance performance differs between age-groups and between different groups of patients. It has not been possible to detect any single balance measure that can predict future fracture [39]. Thus, balance measures, adequate for the age- and patient group, used together with FRAX®, is likely to increase the chance to identify persons at very high risk of sustaining a fall-related fracture. The importance of choosing the adequate balance measure is supported by a previous study showing that poor ability to stand in tandem stance with eyes open doubles the risk of falling [12]. The population median age in that study was 82 years, thus older than the population in our study and the median value for tandem stance with eyes open was 16 s (SD12), compared to 30 s in our study. This suggests that SOLEC may be a more appropriate balance test for the age group included in our study.

The need to use arm support when rising from a chair has previously been used to predict hip fractures in elderly women [40]. The mean age of the population in that study was 7 years higher than in our study. Furthermore, in that study only the need to use arms was registered rather than the time it took to rise five times without support from the arms. Thus, FTSST seems to be an appropriate measure for patients that are older than the participants in our study.

Tandem stance and standing one leg is performed up to 30 s, which means that there is a ceiling effect in those measures. In TSEO, 91.5 % of the participants accomplished 30 s, indicating that this balance measure probably is too easy to detect balance disturbances in this group of patients. Our participants had a median value of 21 s for Walking as fast as possible for 30 m, which respond well to values from other research on similar group of patients [34].

The participants in this study were living at home and had a good self-rated health (mean value 73) which was expected. Hence, other balance measures that have been found appropriate for detecting fall risk among frail elderly, such as the Timed Up and Go test, was not considered suitable [41].

Also, the median FRAX® values among the participants in our study was 0.27 and 0.11 implying low fracture risk, even if all of them had sustained a fall-related fracture and thereby have high risk of fall [11]. This might imply the use of other measures along with FRAX® to improve fracture prediction.

Other fracture risk prediction tools include fall risk in the tool, such as GARVAN and Qfracture. GARVAN has shown to have equal discriminative ability as FRAX® [42] and Qfracture has shown to have better discriminative ability for hip fracture than FRAX® [43]. Both GARVAN and Qfracture include fall risk in the fracture prediction by including history of falls but not balance measures. Hence, by including balance measures in a fracture prediction tool instead of history of falls, it might be possible to identify individuals at high risk of falling even before the first fall.

There are only five men in this study and therefore not possible to adjust for sex. However, when analyzing the results with men excluded, the results were the same. Other authors have adjusted for age when analyzing the correlation between FRAX® and balance measures and found that age explained the correlation [25]. We did not adjust for age in our analysis, since age is included in the FRAX® algorithm and therefore inevitable seems to explain any correlation.

### Strengths and limitations

Limitations of the present study include the missing data entries when calculating the FRAX® values with the online calculation tool. We suspect that the missing data have diluted our results and that correlations would be even stronger without missing data. Another limitation is the absence of bone density in the calculation of FRAX®. However, research in the effect of inclusion of bone mineral density in the FRAX® calculation is inconclusive: inclusion has been shown to both underestimate [40] and overestimated [44] risk. Also, all participants had in fact sustained a fall-related wrist-fracture. Previous fracture indicates a substantial risk of future fracture [45]. Hence, we hope that this means that we have identified persons at a very high risk of fall but it can also mean that the sample is biased, which will have to be considered when reflecting on the results.

### Future research and clinical implications

The results indicate a relationship between FRAX® and clinical balance measures, with the strongest correlation between FRAX® and SOLEC. These findings may be used in future hypothesis testing, where this group is followed for observation of actual fracture incidence thus potentially confirming a high risk group of acquiring fracture. However, the choice of balance measure, adequate for the age- and patient-group, seems crucial, both in research and in clinical practice.

## Conclusion

This study showed that there is an association between clinical balance measures and FRAX®. Hence, the use of clinical balance measures and FRAX® in combination, might improve the identification of individuals with high risk of falls and thereby following fractures. Results enable healthcare providers to optimize treatment and prevention of fall-related fractures.

### Ethics approval and consent to participate

The study was approved by the regional ethical review board in Lund [number 585/2008]. All patients gave their informed consent before entering the study.

### Consent for publication

Not applicable.

### Availability of data and materials

Data supporting findings in the study can be requested from corresponding author.

## Abbreviations

FRAX: Fracture Risk Assessment Tool
FTSST: Five times sit-to-stand test FTSST.
SOLEC: Standing one leg eyes closed
SOLEO: Standing one leg eyes open
TSEC: Tandem standing eyes closed
TSEO: Tandem standing eyes open
